# Effects of body mass index on propofol-induced cardiovascular depression in the Pakistani population

**DOI:** 10.12669/pjms.39.2.6787

**Published:** 2023

**Authors:** Uzma Naeem, Akbar Waheed, Yasmeen Azeem, Muhammad Nazir Awan

**Affiliations:** 1 Dr. Uzma Naeem, M.Phil., Department of Pharmacology, Islamic International Medical College, Riphah University, Rawalpindi, Pakistan; 2 Dr. Akbar Waheed, Ph.D, Department of Pharmacology, Islamic International Medical College, Riphah University, Rawalpindi, Pakistan.; 3 Dr. Yasmeen Azeem, FCPS., Department of Anesthesia, Holy Family Hospital, Rawalpindi, Pakistan; 4 Dr. Muhammad Nazir Awan, FCPS., Department of Anesthesia, Railway General Hospital, Rawalpindi, Pakistan

**Keywords:** Propofol, Body mass index, Cardiovascular effects, Pakistani Population

## Abstract

**Objective:**

To determine the relationship between the patient’s Body Mass Index (BMI) and the cardiovascular effects produced by propofol at a dose of 1.5 mg/kg in the Pakistani population.

**Methods:**

This descriptive cross-sectional study was conducted in the Holy Family Hospital Rawalpindi from August 2021 to January 2022. According to their BMI, one hundred twenty Pakistani individuals 18 to 60 years of age were equally divided into three groups. Group N (n = 40) with a BMI of 18 to 24.9, group OW (n=40) with a BMI of 25 to 29.5, and group O (n=40) with a BMI of 30 to 34.9 were randomized to receive propofol injections at a 1.5 mg/kg dose for induction of anesthesia. We measured mean blood pressure before the propofol and then at one, three, and ten minutes after the injection. Data were analyzed by using SPSS 22.

**Results:**

Mean blood pressure decreases significantly in all groups, as shown by p-values of <0.001 for the first two readings. In group N, blood pressure returned to near normal within ten minutes (p-value 0.061), but in groups, OW and O, mean blood pressure was significantly lower even after ten minutes (p-values 0.005 and 0.001, respectively). Individual variations in propofol response were also observed.

**Conclusion:**

In the Pakistani population, propofol at an induction dose of 1.5 mg/kg to patients with different body weights produces cardiovascular effects with marked standard deviations in each group, which indicate different individual responses.

***Clinical Trial Number:*** NCT05383534 https://register.clinicaltrials.gov/

## INTRODUCTION

Propofol is a phenolic by-product, available as an oil-in-water intravenous emulsion for calming and mesmerizing purposes. It is a broad-spectrum ultrashort-acting anesthetic, which has significant advantages in anesthetic potency and safety.^1^ Easy control of the depth of anesthesia, rapid recovery of consciousness, and less postoperative nausea and vomiting are the characteristics that make propofol a standard agent for induction of anesthesia.^2^,^3^

Common adverse effects of propofol include hypotension, bradycardia, respiratory depression, myoclonus, and pain at the injection site.^4^ The most common among these adverse effects is propofol-induced hypotension; this decrease in blood pressure is due to the inhibition of myocardial contractility, a decrease in peripheral resistance, and sympathetic inhibition.^5^ Regarding the pharmacokinetics of propofol, its highly lipophilic feature leads to a high volume of distribution and a long elimination half-life, although it undergoes extensive metabolism in the liver. Propofol pharmacokinetics and pharmacodynamics are subjected to high interindividual variability, leading to the variabilities in the required induction dose and the adverse cardiovascular effects produced by the propofol.

The factors that mainly contribute to this variability include age, sex, genetic variations, increase in adipose tissue, lean body weight, extracellular fluid, and cardiac output.^6^,^7^ The augmented central volume of distribution and variations in the clearance of drugs affect the plasma concentration of propofol in the obese population because obesity changes body composition and physiology.^7^ It was demonstrated in a previous study that about 40% of the excess mass of obese individuals results from the increased fat-free mass.^8^ Keeping this in mind, it could be assumed that intravenous drug doses scaled according to the total body weight can result in overdosing in overweight and obese individuals and subsequent dose-related adverse effects.

Available studies of weight-related propofol pharmacokinetics are scarce and derived from a small number of patients. We know that no public comparative research compares cardiovascular effects produced by standard propofol doses in patients with different body mass indexes in the Pakistani population.

With this background, this study aimed to investigate the standard propofol dose-induced cardiovascular depression by measuring mean blood pressure in normal, overweight, and obese patients using a population-based approach to predict variabilities related to the same propofol dose. We can minimize the cardiovascular risks associated with propofol anesthesia by predicting these variabilities.

## METHODS

This descriptive cross-sectional study was carried out in Holy Family hospital Rawalpindi’s anesthesia department for six months, from August 2021 to January 2022, collaborating with Islamic International Medical College Rawalpindi. The study procedure was accepted and approved by the institutional ethical review board (Riphah/IIMC/IRC/20/002) and the clinical trial number is NCT05383534. After taking the written and verbal informed consent, one hundred twenty Pakistani patients (both male and female) aged more than 18 and less than 60 were selected for this study. Another essential inclusion criterion was the BMI of patients; 40 patients with normal BMI (Group-N), forty were overweight (Group-OW), and 40 were obese (Group-O). Patients of extreme age and fitted in classes III, IV, V &, and VI of the ASA (American Society of Anesthesiology) scale were excluded from the study. The patient’s BMI was calculated using the formula BMI = kg/m^2,^ where kg is a person’s weight in kilograms and m^2^ is their height in meters squared. The patients with a BMI of 18.5 to 24.9, 25 to 29.5, and 30 to 34.9 were considered normal, overweight, and obese, respectively.^9^

The sample size for this study was estimated by employing a previously published study.^10^ All these patients were in classes I and II of the American Society of Anesthesiologists (ASA)^11^ scale and received Propofol at a dose of 1.5 mg/kg for induction of anesthesia.^12^ The first reading of the mean blood pressure of patients under study was measured before the propofol induction and after 1, 3, and 10 minutes of propofol injection.^11^ Mean arterial pressure (MAP) was calculated using the formula MAP = DP + 1/3(SP – DP). Here DP is the diastolic blood pressure, and SP is the systolic blood pressure.^13^

### Statistical analysis:

BMI and cardiovascular outcomes in the form of mean blood pressure were recorded for all the patients in the form of excel files. Excel files were brought into the Statistical Package for Social Sciences (SPSS) version 22 for analysis. The normal distribution of data was confirmed with the Kolmogorov–Smirnov test, and differences in the mean blood pressure between the three groups at 0, 3, and 10 minutes were analyzed using Student’s paired t-tests with P<0.05 considered significant. All values are given as the mean (±SD). Comparison among the groups was made by Dunn’s All-Pairwise Comparisons Test.

## RESULTS

Our study included 120 Pakistani patients, divided into three groups according to their BMI, N (standard), OW (overweight), and O (obese). Participants’ variables are shown in Table-I.

**Table-I:** Participants’ variables.

Groups	Male	Female	Age ± SD	BMI ± SD
N (n=40)	27	13	45 ± 5.61	21 ± 2.34
OW(n=40)	19	21	49 ± 9.59	27.5 ± 2.11
O (n=40)	11	29	43 ± 7.51	32.5 ± 2.19

Patients of Group-N showed a significant decrease in the mean blood pressure after one and three minutes compared with the pre-propofol reading, with a p-value of <0.001, while the reduction in mean blood pressure was not significant after 10 minutes (p-value 0.061). Within ten minutes of propofol injection, blood pressure comes back to normal in patients with a BMI of 18.5 to 24.9 (Group-N). While in patients with BMI between 25 to 29.9 (Groups-OW & O), we observed that this decrease in blood pressure was significant at three-time intervals with p-values of <0.001, <0.001, and 0.005, respectively for Group-OW, and <0.001, <.001, & <0.001 for Group-O. All these results are summarized in Table-II.

**Table-II:** Comparison of propofol-induced decrease in mean blood pressure among groups.

Groups	BMI of patients	Mean BP before propofol injection		Mean BP after one minute of propofol injection			Mean BP after 3 minutes of propofol injection			Mean BP after 10 minutes of Propofol		

		Mean	SD	Mean	SD	p1	Mean	SD	p2	Mean	SD	p3
Normal (N)	18.5 to 24.9 kg/m^2^	97.800	6.365	86.900	6.644	<.001	90.950	6.304	<.001	96.600	6.067	0.061
Overweight (OW)	25 to 29.9 kg/m^2^	101.050	7.099	83.975	9.960	<.001	95.550	10.869	<.001	98.450	8.819	0.005
Obese (O)	30 to 34.9 kg/m^2^	98.80	8.919	76.125	9.648	<.001	80.200	12.999	<.001	84.525	17.885	<.001

Dunn’s All-Pairwise Comparisons Test: N < OW < O.

**Fig.1:**
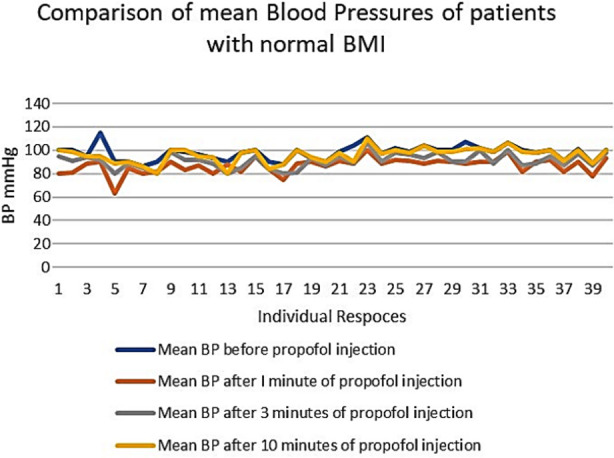
Comparison of mean blood pressures of patients with normal BMI.

**Fig.2:**
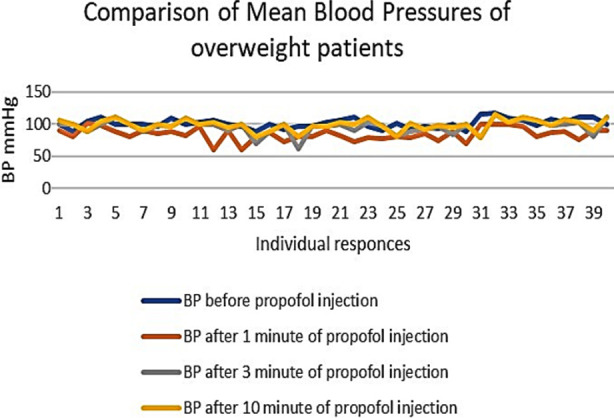
Comparison of mean blood pressures of overweight patients.

**Fig.3:**
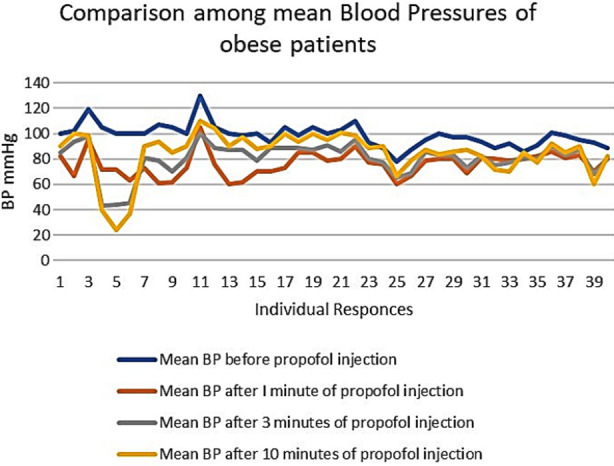
Comparison of mean blood pressures of obese patients.

An important finding of our study was the higher values of standard deviation (SD) among the individuals of the same group, as shown in Table-I. This shows the inter-individual variability or different responses of the individuals with the same BMI and the same dose of Propofol (Fig.1, 2, & 3). These differences are more marked in Group-O (with a BMI of 30 to 34.9), as shown in Fig.3.

## DISCUSSION

In our study, all the participants were Pakistani with no discrimination of sex. The mean dosage of propofol used to induce the anesthesia was 1.5 mg/Kg, the standard dose usually used for induction of anesthesia associated with minimum adverse effects.^12^,^14^ The fluctuations of mean blood pressure in our study were more marked in Group-O, less in Group-OW, and lesser in Group-N. The cardiovascular effects of propofol have been studied by many researchers, but very few of them explained its relationship with BMI in this way.

Our findings of group N are consistent with a recent study from the United States of America using a propofol dose range of 1.5-2.5 mg/kg, which reported hypotensive effects of propofol at 45 seconds and three minute intervals in patients with normal BMI (p-value less than 0.05).^15^ Another study that Kawasaki and his colleagues conducted showed the association of age with hemodynamic fluctuations in patients with normal BMI and observed significant cardiovascular depression.^16^ Recently, in 2022, Oda reported the hypotensive effects of propofol in patients with intellectual disabilities.^17^

According to our knowledge, only one study was conducted in South Africa comparing normal weight, overweight, and obese individuals to observe propofol’s induced cardiovascular effects. But they used an adjusted body mass scaler and didn’t mention the time intervals at which different observations were made.^18^ In our study, the most marked mean blood pressure decrease was observed in Group-O at three-time intervals with p-values of <0.001. These findings are consistent with the results of Ding and Hu, who studied the efficacy and safety of propofol in obese patients and observed a significant decrease in mean arterial pressure (p-value was 0.03).^19^ A comprehensive study on the effects of BMI on propofol pharmacokinetics and dynamics was done by Dong and his colleagues in 2016, but they didn’t compare different BMIs and meant blood pressure monitoring for 10 minutes.^5^ Alam et al. and Khattak et al. from Pakistan used midazolam along with propofol to decrease its adverse effects in cirrhotic and non-cirrhotic patients.^20^ In 2019 safety of propofol was studied in Pakistan and the results showed a less marked decrease in blood pressure after induction with propofol 1 mg/kg.^21^ Hamid and his colleagues tried to use the priming principle to decrease the induction dose of propofol because propofol dose increment is directly related to cardiovascular depression. 22 The incidence of post-induction hypotension was also studied recently in 2022 and it was concluded that induction with propofol & thiopental and orthopedics surgery are the independent risk factors for this hypotension.^23^

The most remarkable finding of our study was the higher values of standard deviations in each comparison. These deviations indicate the individual differences in propofol handling, which affect its serum levels and adverse effects. The most common cause of these variations is the difference in the alleles of the enzymes responsible for the metabolism of propofol.^24^,^25^ To our knowledge, there has been no study from the Pakistan population to evaluate propofol’s effectiveness and adverse effect profile.

### Strengths of the study:

The potential strengths of our study were the adjustment of a single per kg dose for all the patients, dividing them into three groups according to their BMI, and close monitoring of cardiovascular effects for ten minutes. Our findings will help to minimize the cardiovascular adverse effects of propofol.

### Limitations of the study:

We also acknowledge the potential limitations of our study, including the comparison with other doses of Propofol, evaluation of the population’s genetic makeup, and its correlation with the propofol serum levels.

## CONCLUSION

Propofol administration based on body weight in overweight and obese patients is associated with a high risk of overdosing and dose-related adverse effects. Several mass scalars, including the ideal body weight and lean body weight, have been introduced to calculate drug dosages more correctly for overweight and obese patients; however, it is essential to carefully monitor the effects and side effects of the drugs during administration. At an induction dose of 1.5 mg/kg when given to Pakistani patients (with different body weights), Propofol produces cardiovascular effects with marked standard deviations in each group, which indicate different personal responses apart from the body weight effects.

### Future recommendations:

a comprehensive study on the correlation between the propofol serum levels and different alleles of the enzymes responsible for the metabolism of Propofol should be performed.

### Authors Contribution

**UN** conceived, designed, and did statistical analysis & manuscript writing, and is responsible for research integrity and she is the **corresponding author**.

**YA** did data collection

**NA** did a review and final approval of the manuscript.

### Abbreviation:

**BMI:** Body Mass Index

**OW:** Overweight

**N:** Normal

**ICU:** Intensive Care Unit

**O:** Obese

**MAP:** Mean Arterial Pressure

**ASA:** American Society of Anesthesiologists

**DP:** Diastolic Pressure

**SP:** Systolic Pressure

**SPSS:** Statistical Package for Social Sciences

**SD:** Standard Deviation
